# Gastric Varices: First You Have to See Them

**DOI:** 10.1155/1994/32832

**Published:** 1994

**Authors:** Shorland W. Hosking, Alan G. Johnson

**Affiliations:** University Surgical Unit Royal Hallamshire Hospital Sheffield S 10 2 JF UK


decompression with small diameter PTFE interposition grafts.

The confirmation of Sarfeh's experience of durable patency with the use of small diameter ringed PTFE interposition prosthetic grafts appears to have allayed residual reservations about this procedure. Further experience over more extended periods will be required to establish whether it will truly match the excellent long term patency rate and functional results of the DSRS. In my view it has established a place in the operative therapeutic armamentarium for portal hypertension, especially for patients where a DSRS is impossible, for example patients who have undergone a previous ill advised splenectomy. The technical simplicity and safety of this procedure are also appealing in the less common context of emergency shunting, although it is doubtful whether it will have a major impact on the much greater operative mortality experienced with all forms of emergency operative intervention in Childs grade C patients.

*References*

1. Sarfeh, I. J., Rypins, E. B. and Mason, G. R. (1986) A systematic appraisal of portacaval H-graft diameters. *Ann. Surg.*, **204**, 356–63.
2. Johansen, K. (1992) Prospective comparison of partial versus total portal decompression for bleeding esophageal varices. *Surg. Gynecol. Obstel.*, **175**, 528–34.
3. Myburgh, J. A. (1990) Selective shunts: the Johannesburg experiences. *Am. J. Surg.*, **160**, 67–72.
4. Warren, W. D., Millikan, W. J. and Henderson, J. M. *et al.* (1984) Selective variceal decompression after splenectomy or splenic vein thrombosis with a note on splenopancreatic disconnection. *Ann. Surg.*, **199**, 694–72.
5. Myburgh, J. A. (1991) The Warren shunt: Effect of alocoholism on portal perfusion. Discussion on paper by Kawasaki, S. *et al. HPB Surgery*, **5**, 70–73.
6. Johansen, K. H., Girod, C., Lee, S. S. and Lebrec, D. (1990) Mesenteric venous stenosis reduces hyper-ammonemia in the portacaval shunted rat. *Eur. Surg. Res.*, **22**, 170–74.

J. A. Myburgh
Professor of Surgery
University of the Witwatersrand
Medical School
York Road
Parktown, Johannesburg
2193, South Africa

### GASTRIC VARICES: FIRST YOU HAVE TO SEE THEM

### ABSTRACT

*Sarin, S. K., Lahoti, D., Saxena, S. P., Murthy, N. S., and Makwana, U. K. (1992) Prevalence, Classification and Natural History of Gastric Varices: A longterm follow-up study in 568 portal hypertension patients. Hepatology, 16, 1343–1349.*

**To determine the prevalence and natural history of gastric varices, we prospectively studied 568 patients (393 bleeders and 175 nonbleeders) with portal hypertension (cirrhosis in 301 patients, noncirrhotic portal fibrosis in 115 patients, extrahepatic portal vein obstruction in 117 patients and hepatic venous outflow obstruction in 35 patients). Primary (present at initial examination) gastric varices were seen in 114 (20%) patients; more were present in bleeders than in nonbleeders (27% vs. 4%, respectively; $p < 0.001$). Secondary (occurring after obliteration of esophageal varices) gastric varices developed in 33 (9%) patients during follow-up of $24.6 \pm 5.3$ mo. Gastric varices (compared with esophageal varices) bled in significantly fewer patients (25% vs. 64%, respectively). Gastric varices had a lower bleeding risk factor than did esophageal varices ($2.0 \pm 0.5$ vs. $4.3 \pm 0.4$, respectively) but bled more severely ($4.8 \pm 0.6$ vs. $2.9 \pm 0.3$ transfusion units per patient, respectively). Once a varix bled, mortality was more likely (45%) in gastric varix**

**patients. Gastric varices were classifed as gastroesophageal or isolated gastric varices. Type 1 gastroesophageal varices (lesser curve varices) were the most common (75%). After obliteration of esophageal varices, type 1 gastroesophageal varices disappeared in 59% of patients and persisted in the remainder; bleeding from persistent gastroesophageal varices was more common than it was from gastroesophageal varices that were obliterated (28% vs. 2%, respectively; $p < 0.001$). Type 2 gastroesophageal varices, which extend to greater curvature, bled often (55%) and were associated with high mortality. Type 1 isolated gastric varices patients had only fundal varices, with a high (78%) incidence of bleeding. Type 2 isolated gastric varices were mainly (86%) ectopic secondary gastric varices that bled only rarely (9%). Sclerotherapy was more effective in controlling acute bleeding and obliterating varices in gastroesophageal varices than in isolated gastric varices; the latter often required surgery. In conclusion, gastric varices are a common and serious complication of portal hypertension. Our classification was helpful in understanding the natural history and management of gastric varices. (HEPATOLOGY 1992; 16:1343–1349.)**

KEY WORDS: Portal hypertension, gastric varices, variceal bleeding

### PAPER DISCUSSION

Gastric varices attracted little interest until injection sclerotherapy became used widely. Previously, many patients with portal hypertension who bled from any varix would undergo shunt surgery irrespective of the site of bleeding. With the widespread adoption of sclerotherapy, precise identification of the bleeding point became essential, but the effectiveness of sclerotherapy in controlling bleeding from oesophageal varices was not seen when applied to gastric varices. Imprecise terminology and limited, almost anecdotal reports did not help. Fortunately, there have been several studies in the last five years which have made serious attempts to document the prevalence, distribution, bleeding risk and treatment strategies of gastric varices. Sarin and colleagues have written such a paper.

In their study, gastric varices (GVs) were classified along simila̲ir lines to those proposed by ourselves[1] and Korula *et al.*[2]. Five hundred and sixtyeight patients with portal hypertension underwent endoscopy by two independent observers. Gastric varices were recorded when both observers agreed on their presence and distribution. Four main types were described:

1) Lesser curve GVs which were an extension of oesophageal varices (OVs)
2) Fundal GVs which were continuous with OVs
3) Fundal GVs with no OVs
4) Antroduodenal GVs with no OVs.

Twenty per cent of their patients had GVs. The vast majority of GVs (76%) were located on the lesser curve. Treatment of GVs was reserved until they bled but all OVs, whether they had bled or not, underwent sclerotherapy. After injecting OVs, Sarin and colleagues reported that many GVs, disappeared, a few appeared and the remainder were unchanged. Of greater clinical importance is that 12% of lesser curve GVs and 55% of fundal GVs bled: almost half of those who bled died. Such mortality is considerably more than that of bleeding OVs.

This paper confirms some of what has been reported already but raises another very important issue, that of the considerable variation seen in series on GVs. For example, gastric varices have been generally reported in 10–36% of patients who have OVs (some series 0–100%), the prevalence of lesser curve GVs is reported as 4–35% of patients and that of secondary GVs appearing after OV sclerotherapy as 9–30%. These variations deserve closer examination.

Portal hypertension is a progressive condition. The point at which a patient is examined for GVs (and OVs or any other varix) therefore becomes all important. Sarin *et al.*, report that 69% of their cohort had a bleeding history but the site (cause) is not stated. Hence the statement that GVs were more common in patients who had bled does not allow us to stage patients accurately in their chronology of portal hypertension. The remaining 31% had not bled. This problem of heterogeneity creeps into many series and makes interpretation of prevalence figures very hard to compare from one series to another. We know that the aetiology of portal hypertension is not important to the development of OVs and this seems to be true for GVs. Severity of liver disease may be important and is an unfortunate omission from this paper.

How is a GV diagnosed? In theory there should be no difficulty in recognizing a GV via an endoscope. Unfortunately this is far from easy unlike OVs where observer variation is acceptably small. Sarin *et al.*, make a serious attempt to overcome this problem by use of 2 observers. Such a technique might, if anything, underestimate the presence of GVs although we cannot estimate by how much as observer variation was not stated. Attention is rightly drawn to the necessity of full insufflation of the stomach and also to the particular problems of endoscopic examination immediately after a bleed. One wonders whether all observers routinely examine the fundus once a bleeding OV has been diagnosed particularly when the stomach is full of blood. Others have advocated splenoportography rather than endoscopy to demonstrate GVs. In a comparative study of splenoportography and endoscopy in 93 patients, there was good correlation between the two methods with respect to lesser curve varices (which are the simplest to diagnose) but endoscopy diagnosed more fundal varices[3]. What splenoportography cannot demonstrate is the proximity of GVs to gastric mucosa, in other words, the likelihood of bleeding. At present simple endoscopic examination is not always sensitive enough to see GVs and the development of techniques to enhance their appearance would be worthwhile. This becomes all important if treatment is to be administered via the endoscope.

What of treatment? A proportion will bleed (25% over 2 years in Sarin's paper) but certain principles have emerged from recent series. Most reports suggest that lesser curve varices respond well to sclerotherapy with reasonable immediate and long term control of bleeding and acceptable complication rates. The approach to bleeding fundal varices is less clear. They bleed more often (55%–75%) than lesser curve GVs (5%–12%) and the results of scleroptherapy are generally dissappointing. Sarin *et al.*, achieved long term control in about half of 17 patients and similar or worse results are reported by others. Temporary control of bleeding followed by something other than sclerotherapy, such as surgery or possibly drugs, seems to be the way forward. The same seems to be true for isolated antral varices although these are uncommon.

A recurring question is what effect sclerosing OVs has on GVs. Sarin *et al.*, provide us with additional data. They found that lesser curve GVs disappeared in 59% of patients after sclerosing OVs and appeared (secondary GVs) in 9% of patients. Interestingly the proportion of primary and secondary GVs that bled was similair. Only 17% of fundal varices disappeared. We reported a similair incidence of secondary GVs (10%) but others report figures of around 50%. Once again observer variation and methods of data collection seem more likely explanations than a true difference. Why OV sclerotherapy should cause some GVs to appear and others to disappear may be related to the direction of blood flow in OVs. Caudad flow will carry the sclerosant into GVs whereas cephalad flow will do the opposite. The location of perforating veins in this junctional area may also influence GV blood flow after sclerosis of OVs[4].

In conclusion several studies now concur that in patients with OVs there are 2 broad groups of GVs (lesser curve and fundal) which differ in prevalence, morphology bleeding risk and reponse to sclerotherapy. Isolated GVs either due to splenic vein occlusion or generalized portal hypertension are rare and data collection will be much more difficult for this group. Sarin and his collegues have made a valuable contribution to our knowledge of GVs particularly with their thorough technique of examination and recording of data. Future studies of GVs would do well to follow the same path.

### *References*

1. Hosking, S. W., Johnson, A. G. (1988) Gastric varices: a proposed classification leading to management. *Brit. J. Surg.*, **75**, 195–196.
2. Korula, J., Chin, K., Young, K., Yamada, S. (1991) Demonstration of two distinct subsets of gastric varices. Observations during a seven-year study of endoscopic sclerotherapy. *Dig. Dis. Sci.*, **36**, 303–309.
3. Mathur, S. K., Dalvi, A. N., Someshwar, V., Supe, A. N., Ramakantan, R. (1990) Endoscopic and radiological appraisal of gastric varices. *Brit. J. Surg.*, **77**, 432–435.
4. McCormack, T. T., Rose, J. D., Smith, P. M., Johnson, A. G. (1983) Perforating veins and blood flow in oesophageal varices. *Lancet*, **ii**, 1442–1444.

Shorland W. Hosking and Alan G. Johnson
University Surgical Unit
Royal Hallamshire Hospital
Sheffield S10 2 JF
England

